# Comparison of the Clinical Efficacy of Herbal, Chlorhexidine, and Normal Saline Mouthwash in the Management of Chronic Gingivitis

**DOI:** 10.7759/cureus.54336

**Published:** 2024-02-16

**Authors:** Rakhee Sinha, Malabika Shil, Bhavya Srivastava, Deepak Narang, Poulami Goswami, Surbhit Singh, Shivakumar G C

**Affiliations:** 1 Oral Medicine and Radiology, Mithila Minority Dental College and Hospital, Darbhanga, IND; 2 Oral and Maxillofacial Radiology, Nidaan Diagnostic Centre, Pune, IND; 3 Oral Medicine and Radiology, Saraswati Dental College and Hospital, Lucknow, IND; 4 Public Health Dentistry, Mithila Minority Dental College and Hospital, Darbhanga, IND; 5 Oral Medicine and Radiology, People’s College of Dental Science and Research Centre, Bhopal, IND

**Keywords:** gingival index, normal saline, herbal mouthwash, chlorhexidine mouthwashes, chronic gingivitis

## Abstract

Background: The incidence of chronic gingivitis, a widespread inflammatory condition of the gums, is considerable across the demographic spectrum, with potential progression to advanced periodontal pathology in the absence of intervention. The objective of this investigation was to conduct a comparative analysis of the clinical effectiveness of various oral rinses in mitigating the symptoms of chronic gingivitis.

Methods: This empirical study was conducted within the confines of the Department of Oral Medicine and Radiology. A cohort of 60 individuals diagnosed with chronic gingivitis, ranging from 18 to 45 years of age and inclusive of all sexes, was systematically selected for participation.

Results: Quantitative analysis yielded data indicating that the mean score on the gingival index was minimally recorded for participants utilizing herbal mouthwash (HO), in contrast to those administered with normal saline (NS), which displayed the highest mean score. A corresponding trend was observed with the plaque index, where the HO users exhibited the lowest mean values, as opposed to the NS cohort, which demonstrated the highest.

Conclusion: Employing post-hoc statistical evaluations, a pronounced disparity in the mean gingival index was discerned favoring the chlorhexidine (CHX) and HO groups over the NS group. No statistical significance was detected in the comparative mean gingival index between the CHX and HO cohorts. This pattern of findings was paralleled in the plaque index assessments, where the NS group's values were significantly elevated relative to those of both the CHX and HO groups.

## Introduction

Periodontitis is a widespread inflammatory condition that primarily results from microbial biofilms stuck to dental surfaces. Chronic gingivitis represents the initial stage of this condition, characterized by gingival inflammation without the loss of connective tissue or bone. If not addressed, it can evolve into periodontitis, a more destructive entity that may result in tooth exfoliation and systemic health repercussions [1].

The prevailing strategy for managing gingival inflammation hinges on meticulous oral hygiene practices designed to disrupt and eliminate dental biofilms. Mechanical debridement, primarily via toothbrushing and interdental cleaning, constitutes the foundational approach to plaque removal. Nevertheless, mechanical methods may not always achieve comprehensive biofilm eradication, necessitating additional strategies to attenuate plaque accumulation [2-4].

Chemotherapeutic interventions have been explored to augment mechanical debridement. The gold standard for the chemical prevention of gingivitis due to plaque has been chlorhexidine (CHX), a cationic bis-biguanide that kills a variety of microbes [5]. However, adverse side effects like mucosal erosion, dental discoloration, and taste disturbance limit its long-term application [6].

In light of these limitations, there is an ongoing effort to identify alternative chemotherapeutic agents. Herbal mouth rinses have garnered attention, offering a plethora of bioactive phytocompounds with demonstrated anti-inflammatory and antimicrobial activities [7]. Public perception leans toward these botanical products, often favoring them over their synthetic counterparts owing to their perceived natural origin and reduced side effects [8].

Additionally, normal saline (NS), an isotonic sodium chloride solution, though less potent as an antimicrobial agent, has been recognized for its mild antiseptic properties. It serves as a benchmark in clinical trials investigating mouthwash efficacy due to its safety, accessibility, and affordability [9]. Its role in oral health, particularly in the management of gingival inflammation, is underscored by its benign nature, which is devoid of adverse effects and warrants further exploration [10-14].

The goal of this study is to carefully compare how well a herbal formulation works against CHX and NS in treating chronic gingivitis. The ultimate goal is to find the best method that combines effectiveness with the fewest possible side effects in terms of the plaque index and gingival index, respectively.

## Materials and methods

Study design

The study was conducted retrospectively using patient records from the Department of Oral Medicine and Radiology at Mithila Minority Dental College and Hospital in Darbhanga, Bihar, India. This facility served as the primary site for recruitment, treatment, and follow-up of participants. The Institutional Review Board (IRB) assigned the ethical clearance number "IEC/MMDC/2023/08/02" to this investigation after a thorough review.

Sample size

A total of 60 subjects, who had been previously diagnosed with chronic gingivitis, were selected for inclusion in the study. These subjects were chosen based on their attendance at the specified department within the dental college and hospital.

Selection criteria

The selection process involved stringent inclusion and exclusion criteria to ensure the homogeneity of the study population. Patients between 18 and 65 years of age and those without any systemic disorders were considered for inclusion in this study. Subjects were excluded if they presented with advanced periodontal inflammation or severe malalignment of teeth, which could interfere with the accurate measurement of indices or the efficacy of mouthwashes. Additionally, individuals with hypersensitivity to any of the mouth rinse components were excluded to prevent adverse reactions. Pregnant females and nursing mothers were not considered for the study to avoid any potential risks to them or their children. Finally, patients who were unwilling to complete the treatment protocol or those with known systemic diseases that could affect the study's outcomes were also excluded.

Procedures

**Figure 1:**
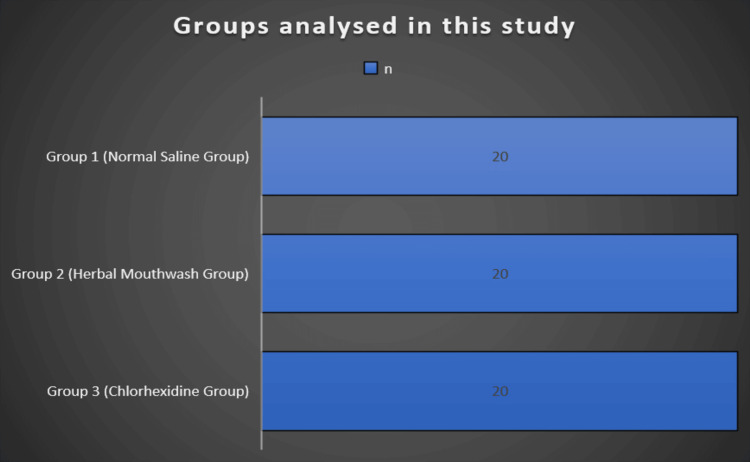
Groups analyzed in this study (n being the number of participants)

Upon selection, the subjects were divided retrospectively into three distinct groups for analysis. Each group consisted of 20 subjects, and assignment to each group was based on the type of mouthwash treatment they had received in conjunction with oral prophylaxis (Figure 1). This sample size was drawn upon in accordance with a previous investigation done across similar parameters and objectives as ours [15].

Clinical indices recorded

Two primary indices were recorded for each subject at the beginning of the study (baseline, 0th day) and after five days of treatment (fifth day). These were:

1. The approximal plaque index, which measures the presence and severity of dental plaque.

2. The gingival index, as defined by Loe and Silness, assesses the severity of gingivitis based on the condition of the gingiva.

The data collected at these two time points were used for subsequent comparison and analysis to evaluate the clinical efficacy of each mouthwash in managing symptoms of chronic gingivitis.

Statistical analysis

For intergroup comparisons, an analysis of variance (ANOVA) test was employed to detect any significant differences in the mean scores of the gingival and plaque indices among the three groups. In cases where the ANOVA test indicated significant differences, a post hoc test (such as Tukey's HSD [honestly significant difference] test) was utilized to identify which specific groups differed from each other. The level of significance was set prior to analysis. A p-value of less than 0.05 was considered to indicate a statistically significant difference between the means of the groups being compared.

## Results

**Figure 2:**
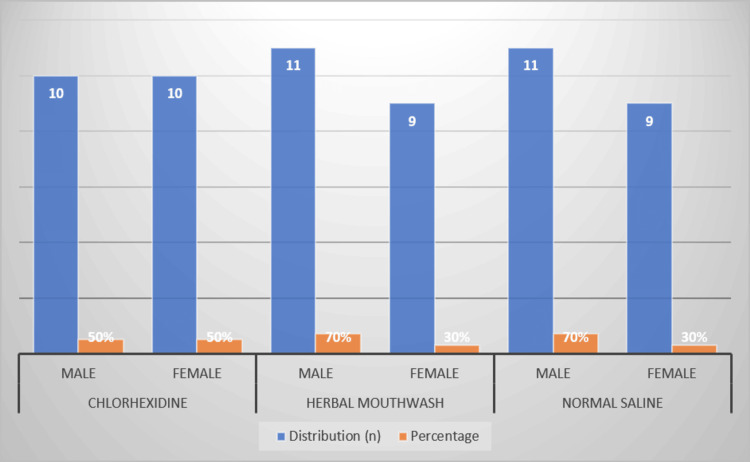
Gender distribution across the groups in terms of the analyzed groups

Table 1 and Figure 2, respectively, show the gender distribution among the study groups. Each group had an equal number of males and females in the CHX group, while the herbal mouthwash (HO) and NS groups had a higher percentage of males (70%) compared to females (30%).

**Table 1:** Gender distribution among the assessed groups

Group	Gender	Distribution
Chlorhexidine	Male	10 (50%)
	Female	10 (50%)
Herbal mouthwash	Male	11 (70%)
	Female	9 (30%)
Normal saline	Male	11 (70%)
	Female	9 (30%)

Table 2 presents the comparative analysis of the gingival and plaque indices across the three study groups. The CHX group had mean gingival and plaque index scores of 0.70 and 0.80, respectively, with the HO group closely following with means of 0.66 for the gingival index and 0.77 for the plaque index. In contrast, the NS group had significantly higher means of 1.59 for the gingival index and 1.86 for the plaque index. The standard deviations showed that the groups were not all the same, but the differences between them were statistically significant (p<0.0001). The NS group had significantly higher index scores than the CHX and HO groups, which were not significantly different from each other.

**Table 2:** Plaque and gingival index comparative analysis SD, standard deviation; NS, normal saline; CHX, chlorhexidine; HO, herbal mouthwash.

Index	Group	Mean	SD	p-Value	Post hoc test results
Gingival index	Chlorhexidine	0.70	0.25	p<0.0001	NS>CHX>HO; Significant
	Herbal mouthwash	0.66	0.16		
	Normal saline	1.59	0.55		
Plaque index	Chlorhexidine	0.80	0.31	p<0.0001	NS>CHX>HO; Significant
	Herbal mouthwash	0.77	0.30		
	Normal saline	1.86	0.61		

Table 3 provided additional insight with post hoc test details, which further analyzed the pairwise differences between groups. The comparisons of CHX vs. NS and HO vs. NS were both significant for the gingival index (p=0.0001). However, there was no significant difference between CHX and HO for both the gingival and plaque indications (p=0.918 and 0.94, respectively), indicating that these two treatments were similarly effective.

**Table 3:** Post hoc test details for gingival and plaque indices

Index	Comparison	p-Value	Significance
Gingival index	Chlorhexidine vs herbal mouthwash	0.918	Not significant
	Chlorhexidine vs normal saline	0.0001	Significant
	Herbal mouthwash vs normal saline	0.0001	Significant
Plaque index	Chlorhexidine vs herbal mouthwash	0.94	Not significant

Table 4 details the ANOVA for the comparative analysis of the gingival and plaque indices. This statistical test was used to determine if there were significant differences between the groups. The between-group variation was significant for both indices, with F values of 26.78 for the gingival index and 30.20 for the plaque index and p values less than 0.0001, indicating a high level of statistical significance.

**Table 4:** ANOVA for comparative analysis of gingival and plaque indices ANOVA, analysis of variance.

Index	Source of variation	Sum of squares	Degrees of freedom	Mean square	F-value	p-Value
Gingival index	Between groups	2.30	2	1.15	26.78	<0.0001
	Within groups	1.20	57	0.021		
Plaque index	Between groups	3.10	2	1.55	30.20	<0.0001
	Within groups	1.52	57	0.027		

Table 5 provided the significance testing results, showing the mean differences between groups for both indices, along with their standard errors and 95% confidence intervals. The mean difference was 0.89 for the gingival index and 1.06 for the plaque index, both with p-values less than 0.0001, confirming the significant differences observed in Table 1.

**Table 5:** Significance testing for gingival and plaque indices CI, confidence interval.

Index	Mean difference	Standard error	95% CI	p-Value	Significance
Gingival index	0.89	0.08	(0.73, 1.05)	<0.0001	Significant
Plaque index	1.06	0.09	(0.88, 1.24)	<0.0001	Significant

## Discussion

The data analysis showed that both the gingival and plaque indices had high F statistics and p values that were below the 0.0001 level needed to show a statistically significant difference between the experimental groups. After more research, it was found that the groups treated with CHX and the herbal formulation did better than the group treated with NS. There was no clear difference in how well CHX and the HO worked as a treatment. The implications of these findings are multifaceted concerning the evolution of oral hygiene protocols. Given that the herbal formula worked just as well as CHX, it is possible that herbal concoctions could become useful alternatives to CHX, which could lower the risk of side effects that come with long-term use of CHX. Such insights could instigate a reevaluation of prevailing guidelines for routine oral care and chronic gingivitis management. The information also makes it more important to keep looking into botanical solutions, which might offer a natural, effective, and all-around approach to dental health.

NS does not seem to be very useful as a treatment for changing gingival and plaque levels. This means that it is more useful as a control or baseline comparison than as an effective treatment method. So, this study lays the groundwork for future research that could further isolate and identify the active phytochemical components in HOs that are responsible for their anti-inflammatory effects. This could lead to the creation of new, better oral healthcare products. In line with our results, comparative literature like the study by Narayan and Mendon [3] shows that Triphala, Hi Ora, and CHX significantly reduce the formation of new plaque more effectively than some commercial mouthwashes, like Colgate Plax. These parallels lend credence to the assertion that CHX and specific herbal formulations exhibit anti-plaque capabilities.

In contrast, Parwani et al. [4] contend that HO and 0.2% CHX are the most effective anti-plaque agents, both outperforming regular saline in terms of effectiveness. Our results are in line with this order of effectiveness, though there is a big difference in the statistical significance between the herbal and saline groups, which is different from what Parwani et al. [4] said, which was that there was no difference. A deviation from our research focus is observed in the study by Ravikumar et al. [12], which delves into the in vitro antifungal efficacy of various mouthwashes against *Candida albicans*. Their results that Hi-Ora regular mouthwash is better than Hi-Ora sensitive and CHX in this way are not directly related to our clinical endpoints, but they do support the main idea of our study, which is that herbal formulations may be strong alternatives to CHX [13,14].

In this study, a botanical mouthwash worked just as well as CHX at reducing gingival and plaque levels. This supports the results of Mali et al. [2], who found that over three weeks, there was not much difference between the effects of CHX and a turmeric-based rinse on these levels. In the same way, Deshmukh et al. [8] found that CHX, a botanical rinse (Hi-Ora), and a probiotic rinse all worked about the same in stopping plaque formation, keeping the gums healthy, and maintaining good oral hygiene. These results collectively infer that botanical solutions can rival synthetic mouthwashes in oral health management.

Contrastingly, the results from additional literature reveal deviations from our findings. Talebi et al. [13] inferred a greater antifungal efficacy in synthetic mouthwashes compared to their botanical counterparts, with Oral B demonstrating the highest potency against *C. albicans*. This suggests that the antifungal properties may be more pronounced in chemical-based formulations. Pathan et al. [15] observed that CHX exhibited superior antimicrobial activity against specific bacterial strains via agar dilution assays, though no such difference was noted for other bacterial strains. This implies that the effectiveness of mouthwashes may be contingent upon the microbial species and the experimental approach employed.

Our methodology did not incorporate enzymatic assays such as BAPNA, which Mali et al. [2] utilized for quantifying the enzymatic activity of periodonto-pathogenic microorganisms. The absence of such biochemical evaluation in our study might signal a gap, as it offers an alternate lens to gauge mouthwash efficacy, focusing on molecular markers rather than solely clinical outcomes.

Janakiram et al. [16] synthesized data from 24 randomized controlled trials encompassing 1,597 participants, comparing the efficacy of herbal toothpaste (HTP) and herbal mouth rinse (HMR) against their non-herbal counterparts. The synthesis revealed that HTP exhibited superior plaque reduction when compared to non-herbal toothpaste (standard mean difference [SMD] 1.95, 95% confidence interval [CI] 0.97-2.93). Conversely, non-herbal mouth rinses demonstrated enhanced long-term efficacy relative to HMR (SMD -2.61, 95% CI 4.42-0.80). HTP was comparable to fluoride toothpaste and surpassed non-fluoride variants in plaque mitigation. Nevertheless, these conclusions were drawn from studies deemed to have low methodological robustness.

Suresh et al. [11] reviewed seven RCTs to evaluate the comparative effectiveness of herbal and traditional toothpastes. The analysis concluded that HTPs, particularly those infused with green tea extracts, matched the plaque-reducing and anti-gingivitis efficacy of conventional toothpastes, including those containing fluoride. Yet, the review underscored the necessity for prolonged-duration studies to substantiate these preliminary findings.

Based on the observed outcomes of this study, several recommendations can be formulated for clinical practice and further research. Given the similar efficacy of herbal and CHX mouthwashes in reducing gingival and plaque index scores, it is recommended that HOs be considered as a viable adjunct to mechanical plaque removal for the management of chronic gingivitis, particularly for patients who may experience adverse effects from CHX or prefer natural alternatives. Healthcare professionals should be encouraged to remain abreast of emerging evidence regarding the efficacy and safety profiles of various mouthwash formulations and to tailor their recommendations to individual patient needs and preferences. For patients with chronic gingivitis, a regimen that includes the use of an effective mouthwash could be beneficial as part of a comprehensive oral hygiene program. For future research, it is recommended that studies be designed with larger sample sizes to confirm the generalizability of the results. Additionally, the formulation of HOs should be standardized to ensure the consistency and reproducibility of research findings. Investigations into the long-term effects of these mouthwashes on oral health would be valuable, as would studies that control for confounding lifestyle factors. It is also recommended that future studies include a true placebo control group to discern the specific therapeutic effects of the active mouthwash ingredients. Such research could help to further validate the clinical utility of mouthwash as an adjunct to conventional oral hygiene practices and could potentially lead to the development of new formulations with optimized efficacy and reduced side effects.

The study, while providing valuable insights into the efficacy of different mouthwash treatments for chronic gingivitis, was not without limitations. First, the sample size, comprising 60 patients, was relatively modest, which could potentially limit the generalizability of the findings to a broader population. A larger sample size would be beneficial for enhancing the statistical power of the study and providing a more robust assessment of the mouthwashes' efficacy. Secondly, the duration of the study was limited to a short-term observation period. Longitudinal effects and the sustainability of the observed benefits over an extended period were not evaluated. The long-term efficacy and safety of regular use of these mouthwashes, particularly the herbal formulation, remain to be established. It is also noteworthy that the study did not include a placebo control group; the NS group served as the control. While NS is not an active treatment, a true placebo would lack any potential therapeutic effect, including the mechanical flushing action of a mouth rinse, which could contribute to plaque removal.

## Conclusions

Within the confines of the conducted study, both CHX and the HO Hi-Ora exhibited comparable anti-plaque activity, with the latter demonstrating the absence of side effects. The research yielded several conclusions from its findings. Post-hoc tests revealed that the mean gingival index for the saline group was significantly higher than that of the CHX and HO groups. Furthermore, the analysis indicated no significant difference in the gingival index between the CHX and HO groups, suggesting equivalent efficacy in reducing gingival inflammation. In addition, the mean plaque index was significantly greater in the saline group as compared to the CHX and HO groups, as shown by post hoc testing. This highlighted the superior anti-plaque properties of the mouthwashes as opposed to saline. Correspondingly, no significant difference was noted between the CHX and HO groups regarding the plaque index. This consistency in the indices for both gingival and plaque measures underscores the potential of the HO to be as effective as CHX in managing dental plaque without the associated side effects.
